# Action and Reaction

**Published:** 1891-01

**Authors:** 


### ACTION AND REACTION.

(Proceedings of I. H. A., evening session, June 24th, 1890.)

Dr. Hawley—I have one objection to make to the paper of Dr. Kent. In it he seems to me to be a little mixed in his use of the words action and reaction. He states, if I understand him aright, that all the symptoms resulting from the drug are drug action. Then he speaks frequently of the reaction of the vital force. There is some confusion here, to my mind, which might have been avoided by a little circumlocution.

Dr. Custis—The diarrhœa produced by the Opium must have been due to some idiosyncrasy in the patient, and shows that Opium was not a proper remedy for that person. Different people are subject to different diseases, and the same drug will operate differently upon different constitutions, and hence by a study of the peculiar susceptibilities of our patients we may often get a clue to the class of remedies needed. One man, for instance, will always have rheumatic conditions follow an exposure and he will have a certain class of remedies to which he is most susceptible, and among them you will find his remedy. Some people are so susceptible to certain remedies that they can never take them without producing aggravations ; these are due to the idiosyncrasy of the person and not to the double action of the drug. When a patient is so susceptible to Bell., for instance, that patient will never be helped by Bell., and would be a poor person on whom to prove Bell., because you would not get the finer symptoms. The two fields for study are the nature of the disease and the action of the drug.

Dr. Johnstone—The gentlemen who followed Dr. Kent and preceded me, have hardly criticized the paper, but simply confirmed it. The action of the drug is purely primary, and causes the vital force to react toward health.

The drug has an action, and the body has a reaction ; the drug gives the impulse, the push to the deranged vital force, which causes it to reach toward normal life. Drugs have only one action, and that is always sick-making; it is the body which reacts. Drugs sometimes kill people when very accurately fitted to the symptoms of the case, especially if too frequently repeated. I have had one case of that kind where the patient did not improve under the indicated remedy, and I believe would have lived longer without it.

Dr. Hitchcock—There seems to be difficulty in understanding the terms action and reaction. I cannot see how anything but the reaction of the body can ever be manifest to us. Whether the individual is made sick by a drug or by a natural cause, it is the vital power trying to overcome the disturbing influence that makes symptoms which are the only things of disease that are manifest to us. Hence it is plainly the reaction of the vital force, in all cases that makes symptoms ; and the only thing that we see is reaction and not action.

When we give a remedy to a prover we get certain results ; these results are simply the efforts of the vital force to get rid of or overcome the power which is disturbing it. They are the reaction of the system, not the action of the drugs; of this latter we know nothing, and therefore no line can be drawn between action and reaction. In the case of the hand plunged into cold water, the first effect is entirely mechanical and cannot be compared to the effect of a potency; the after effects are also entirely different. I do not think it is a fair example.

